# Radiation‐induced tumor immunity in patients with non‐small cell lung cancer

**DOI:** 10.1111/1759-7714.13122

**Published:** 2019-06-22

**Authors:** Natalie A. Lockney, Mei Zhang, Christopher G. Morris, Romaine Charles Nichols, Paul Okunieff, Steven Swarts, Zhenhuan Zhang, Bingrong Zhang, Amy Zhang, Bradford S. Hoppe

**Affiliations:** ^1^ Department of Radiation Oncology University of Florida Gainesville USA

**Keywords:** Abscopal effect, lung cancer, radiation therapy, tumor immunity

## Abstract

**Background:**

Radiation‐induced tumor immunity (RITI) influences primary tumor growth and development of metastases in preclinical cancer models with conventional radiotherapy. Antigen‐specific immune responses have also been shown for prostate cancer treated with radiotherapy. We examined whether RITI can be induced in patients with non‐small cell lung cancer (NSCLC) following proton radiotherapy.

**Methods:**

Pre‐ and post‐radiotherapy plasma samples from 26 patients with nonmetastatic NSCLC who received radiotherapy between 2010 and 2012 were evaluated by western blotting for IgG and IgM bands to assess RITI response to tumor antigens from lung cancer cell lines. Statistical analysis was used to evaluate any correlation among IgG or IgM and clinical outcomes.

**Results:**

Twenty‐one patients received proton therapy at 2 GyRBE/fraction (*n =* 17) or 6–12 Gy/fraction (*n* = 4); five received photon therapy at 2–2.5 GyRBE/fraction. Compared with the pretreatment baseline, new IgG or IgM binding was detected in 27% and 50% of patients, respectively. New IgG bands were detected in the 25–37 kD, 50–75 kD, and 75–100 kD ranges. New IgM bands were detected in the 20–25 kD, 25–37 kD, 37–50 kD, 50–75 kD, and 75–100 kD ranges. There was no difference in IgG and/or IgM RITI response in patients treated with photons versus protons, or in patients who received SBRT compared to standard fractionation (*P* > 0.05). There was no difference in overall survival, metastasis‐free survival, or local control based on IgG and/or IgM RITI response (*P* > 0.05).

**Conclusion:**

RITI can be induced in patients with NSCLC through upregulated IgG and/or IgM. RITI response was not associated with proton versus photon therapy or with clinical outcomes in this small cohort and should be examined in a larger cohort in future studies.

## Introduction

Non‐small cell lung cancer (NSCLC) is the leading cause of cancer deaths.^1^ Although surgery and stereotactic body radiotherapy (SBRT) have been associated with good local control (LC) rates between 80% and 90% in patients with stage I NSCLC,^2^, ^3^ prognosis has generally been poor in those with advanced disease treated with radiation therapy (RT) with or without surgery and chemotherapy. While current investigation with RT has been primarily focused on improving LC rates, another area of interest is the indirect anticancer effects of RT on cancers outside the treatment field, or the abscopal effect.^4^

Studies have demonstrated that RT‐induced tumor immunity (RITI) can affect both primary tumor growth and systemic development of metastases in murine models of breast cancer and lung cancer treated with conventional photon radiation.^5^, ^6^ RT using photons is thought to induce cell death via DNA damage and/or membrane‐dependent signaling pathways that consequently lead to apoptosis.^7^, ^8^ An additional method of cell death may occur if RT leads to antitumor immunity by promoting tumor antigen presentation by dendritic cells, which could cause an abscopal effect.^5^, ^6^ In a clinical study, Nesslinger *et al*. reported that external‐beam RT and brachytherapy, but not surgery, of nonmetastatic prostate cancer were associated with the development of treatment‐associated autoantibody responses in 14% and 25% of patients, respectively.^9^

NSCLC lends itself to the study of anti‐tumor immunity owing to its high number of nonsynonymous mutations compared to other tumor types, due to mutagens such as tobacco smoke, thereby providing an avenue for development of anti‐tumor vaccines and immunotherapy.^10^ In the present study, our primary objective was to investigate whether radiation promotes production of anti‐human antibodies in patients with NSCLC treated with either photon or proton irradiation by measuring IgG and IgM expression in patient plasma samples pre‐ and post‐RT in response to tumor antigens. Our secondary objectives were to evaluate if patients with RITI responses experience improved survival rates and if patients treated with proton radiotherapy exhibit increased RITI responses compared to patients treated with photon therapy.

## Methods

### Patient selection

Between 2010 and 2012, 26 patients with nonmetastatic NSCLC were enrolled prospectively on an institutional review board‐approved study at the University of Florida Proton Therapy Institute (UFPTI), which allowed collection and analysis of their blood prior to, during, and following radiation treatment to the lung with either photons or protons. Patient, tumor, and treatment information was collected from the medical record. Chemotherapy was delivered either as induction therapy before RT (*n* = 1), concurrently (*n* = 15), or not at all (*n* = 10). Clinical outcomes recorded included death, development of metastatic disease, or local recurrence.

### Radiation treatment

Patients were treated with photon or proton therapy according to physician practice at our institution as previously described.^11^, ^12^

### RITI analysis

The method used was adapted from Nesslinger *et al*.^9^ Patient plasma samples were collected serially before, during, and after RT and western blot techniques were then used to assess the tumor‐specific RITI. Patient blood samples were collected at the following time intervals: before RT, day 2 of RT, day 14 of RT, day 28 of RT, day 56 of RT, 1 month after RT, and 3 months after RT. Whole blood was collected in EDTA tubes and then centrifuged to separate cells from plasma. Plasma samples were then aliquotted and stored at −80°C.

Eight lung cancer cell lines were used for tumor‐associated antigens for immunoblot analysis: H460 (large cell), H23 (lung adenocarcinoma), H522 (lung adenocarcinoma), H1299 (lung carcinoma), A549 (lung carcinoma), H520 (lung squamous cell carcinoma), H2882 (lung squamous cell carcinoma), and H2170 (lung squamous cell carcinoma). This methodology has been adopted from Nesslinger *et al*.^9^ and successfully used in our own laboratory as part of a similar study in breast cancer. Cell lines were purchased from the American Type Culture Collection (Manassas, VA) and maintained in RPMI‐1640 medium (Sigma‐Aldrich, St. Louis, MO) containing 10% heat‐inactivated FBS (Life Technologies, Grand Island, NY) in a 37°C incubator in a humidified atmosphere of 5% CO_2_. Cell lysate was extracted with RIPA lysis buffer (Thermo Fisher Scientific, Waltham, MA, USA) supplemented with a protease inhibitor cocktail (Sigma‐Aldrich). Total lysate protein was quantified using BCA Protein Assay Kit (Thermo Fisher Scientific).

**Figure 1:**
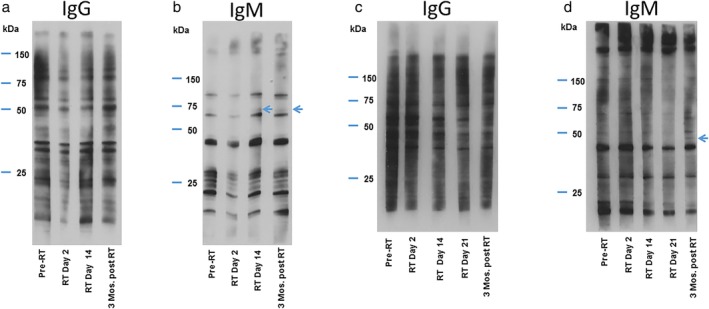
Representative western blot analyses for IgG and IgM radiation‐induced tumor immunity (RITI) response. Panels (**a**) and (**b**) represent western blot analysis of patient serum probed against mixed cell lysates from lung squamous cell carcinoma cell lines H520, H2882, and H2170 for (**a**) IgG and (**b**) IgM for a 75‐year‐old woman with stage IB lung squamous cell carcinoma treated with stereotactic body radiation therapy (RT) with photons to a dose of 60 Gy in 10 fractions. (**a**) No new IgG bands were detected. (**b**) IgM response weighing 50–75 kDa was detected day 14 following RT start and persisted to at least 3 months post‐RT (depicted by arrows). Panels (**c**) and (**d**) represent a western blot analysis of patient serum probed against mixed cell lysates from lung squamous cell carcinoma cell lines H520, H2882, and H2170 for (c) IgG and (**d**) IgM for a 71‐year‐old man with stage IIIA squamous cell carcinoma of the lung treated with proton RT to a dose of 74 Gy (RBE) in 37 fractions with concurrent chemotherapy. (**c**) No new IgG bands were detected. (**d**) New IgM band weighing 37–50 kDa was detected 3 months (Mos) post‐RT (depicted by arrows).

Next, 600–650 μg of protein from cell lysates from the tumor cell lines were separated using 10% Bis‐Tris PAGE gel and transferred to PVDF membrane (Millipore, Bedford, MA) at 90–100 V for 2 h. The membrane was blocked with Tris‐buffered saline, 0.1% Tween 20, and 5% milk for 45 min. The membrane was then cut into strips for each patient. The first antibody was patient plasma diluted 1:1000 in a diluent prepared with Tris‐buffered saline, 0.1% Tween 20, and 1% milk for each available time point. The second antibody was either horseradish peroxidase–conjugated goat anti‐human IgG (Jackson ImmunoResearch, West Grove, PA) diluted 1:20 000 or anti‐human IgM (Jackson ImmunoResearch, West Grove, PA) diluted 1:10 000 in the diluent and visualized by enhanced chemiluminescence (Pierce ECL, Thermo Fisher Scientific). Fig. 1 shows a representative western blot analysis for a patient. Note that for patients with lung squamous cell carcinoma, the serum was probed against the aforementioned squamous cell cancer cell lines mixed lysates (H520, H2882, and H2170) while, for patients with lung adenocarcinoma, serum was probed against the nonsquamous lung cancer cell lines (H460, H23, H522, H1299, and A549).

### Statistical analysis

Patients were generally followed with computed tomography (CT) scans every 3 to 4 months for the first 2 years, then every 6 months. Overall survival (OS) was measured from the initiation of RT until death from any cause or last follow‐up. Metastasis‐free survival (MFS) was measured from the initiation of RT until the development of metastatic disease in the contralateral lung or distant site or last follow‐up. LC was defined as the absence of radiographic recurrence within the treatment target and measured from the initiation of RT until the development of a local recurrence or last follow‐up. Statistical analysis was performed with JMP software (SAS Institute, Cary, NC). The Kaplan‐Meier product limit method provided estimates of OS, LC, and MFS. The log‐rank test statistic assessed statistical significance between strata of selected prognostic factors including age, gender, histology, stage, chemotherapy, radiation modality, standard fractionation versus SBRT, IgG response, and IgM response.

## Results

Patient and tumor characteristics are summarized in Table 1. Twenty‐one patients received proton therapy at 2 Gy (relative biological effectiveness; RBE) per fraction (*n* = 17) or 6–12 Gy(RBE)/fraction (*n* = 4), and five patients received photon therapy at 2–2.5 Gy(RBE)/fraction. The median RT dose was 70 Gy in 35 fractions.

**Table 1:** Patient and treatment characteristics with grouping by IgG and IgM RITI response

		IgG RITI response			IgM RITI response		
	All patients, *n* = 26 No. (%)	Positive, *n =* 7 No. (%)	Negative, *n =* 19 No. (%)	*P‐* value	Positive, *n =* 13 No. (%)	Negative, *n =* 13 No. (%)	*P‐* value
Patient age, years, median (range)	67.8 (49.0–90.6)	66.4 (59.8–79.2)	68.0 (49.0–90.6)	0.8810	68.0 (49.0–90.6)	67.7 (58.4–80.6)	0.5557
<70 years	14 (53.8)	4 (57.1)	10 (52.6)	0.9999	7 (53.8)	7 (53.8)	0.9999
≥70 years	12 (46.2)	3 (42.9)	9 (47.4)		6 (46.2)	6 (46.2)	
Patient sex							
Male	9 (34.6)	1 (14.3)	8 (42.1)	0.3574	5 (38.5)	4 (30.8)	0.9999
Female	17 (65.4)	6 (85.7)	11 (57.9)		8 (61.5)	9 (69.2)	
Tumor histology							
Adenocarcinoma	10 (38.5)	4 (57.1)	6 (31.6)	0.6652	5 (38.5)	5 (38.5)	0.6951
Squamous cell carcinoma	14 (53.8)	3 (42.9)	11 (57.9)		8 (61.5)	6 (46.2)	
Adenosquamous	1 (3.8)	0 (0.0)	1 (5.3)		0 (0.0)	1 (7.7)	
Large cell	1 (3.8)	0 (0.0)	1 (5.3)		0 (0.0)	1 (7.7)	
Stage							
IA	1 (3.8)	1 (14.3)	0 (0.0)	0.9999	0 (0.0)	1 (7.7)	0.6728
IB	5 (19.2)	1 (14.3)	4 (21.1)		2 (15.4)	3 (23.1)	
IIA	2 (7.7)	0 (0.0)	2 (10.5)		1 (7.7)	1 (7.7)	
IIB	0 (0.0)	0 (0.0)	0 (0.0)		0 (0.0)	0 (0.0)	
IIIA	12 (46.2)	3 (42.9)	9 (47.4)		6 (46.2)	6 (46.2)	
IIIB	6 (23.1)	2 (28.6)	4 (21.1)		4 (30.8)	2 (15.4)	
Chemotherapy							
Concurrent	15 (57.7)	3 (42.9)	12 (63.2)	0.9999	7 (53.8)	8 (61.5)	0.9999
Induction	1 (3.8)	1 (14.3)	0 (0.0)		1 (7.7)	0 (0.0)	
No	10 (38.5)	3 (42.9)	7 (36.8)		5 (38.5)	5 (38.5)	
Radiation type							
Proton therapy	21 (80.8)	4 (57.1)	17 (89.5)	0.1014	11 (84.6)	10 (76.9)	0.9999
Photon therapy	5 (19.2)	3 (42.9)	2 (10.5)		2 (15.4)	3 (23.1)	
Radiation subtype							
Standard fractionation	22 (84.6)	6 (85.7)	16 (84.2)	0.9999	12 (92.3)	10 (76.9)	0.5930
SBRT	4 (15.4)	1 (14.3)	3 (15.8)		1 (7.7)	3 (23.1)	

RITI, radiation‐induced tumor immunity; SBRT, stereotactic body radiation therapy.

New IgG or IgM binding was detected after RT in seven patients (27%) and 13 patients (50%), respectively. IgG bands were detected in the 25–37 kD range, 50–75 kD range, and 75–100 kD range in three, one, and three patients, respectively. Each of the seven patients had only a single IgG band detected. IgM bands were detected in the 20–25 kD range (two patients), 25–37 kD range (six patients), 37–50 kD range (three patients), 50–75 kD range (five patients), and 75–100 kD range (two patients), with four patients having multiple IgM bands detected. The median time to development of new IgG bands was day 28 of RT (range, day 2 of RT to 1 month after RT), and IgG band persisted to 3 months in four of seven patients (57%). The median time to development of new IgM bands was day 56 of RT (range, day 2 of RT–3 months post‐RT). At the time of last plasma assessment (3 months post‐RT), 10 of 13 patients (77%) had persistent IgM bands. Table 1 shows there were no statistically significant differences in patient, tumor, or treatment characteristics by IgG and IgM band status. Notably, in this small cohort there was no detectable difference in IgG and/or IgM RITI response in patients treated with photons compared to patients treated with protons, or in patients who received SBRT compared to standard fractionation (*P* > 0.05).

The median follow‐up was 36 months (range, 1–70 months). For all patients, actuarial OS, MFS, and LC rates at 2 years were 62%, 49%, and 86%, respectively. The 5‐year OS, MFS, and LC rates were 27%, 34%, and 80%, respectively. As demonstrated in Table 2, stage I/II patients had greater 5‐year OS (50%) compared to stage III patients (17%; *P* = 0.023). Patients with squamous cell carcinoma compared to other histologies (adenocarcinoma, large cell carcinoma, adenosquamous carcinoma) also experienced improved 5‐year OS (43% vs. 8%, *P* = 0.031). There was no difference in 5‐year OS based on IgG and/or IgM RITI response or any other examined clinical factors (*P* > 0.05).

**Table 2:** Kaplan‐Meier analysis for overall survival

Variable	5‐year overall survival rate	*P*‐value
Age at RT, years		0.321
<70	14%	
≥70	42%	
Patient sex		0.214
Female	29%	
Male	22%	
Histology		0.031
SCC	43%	
Other	8%	
Stage		0.023
I/II	50%	
III	17%	
Chemotherapy		0.077
No	40%	
Yes	19%	
RT type		0.982
Proton	29%	
Photon	20%	
IgG response		0.799
Absent	32%	
Present	14%	
IgM response		0.194
Absent	38%	
Present	15%	
IgG or IgM response		0.303
Absent	40%	
Present	19%	

RT, radiation therapy; SCC, squamous cell carcinoma.

Table 3 shows the MFS analysis. Patients with squamous cell carcinoma compared to other histologies experienced improved 5‐year MFS (52% vs. 10%; *P* = 0.022). There was no difference in 5‐year MFS based on IgG and/or IgM RITI response or any other examined clinical factors (*P* > 0.05).

**Table 3:** Kaplan Meier analysis for metastasis‐free survival

Variable	5‐year metastasis‐free survival rate (%)	*P*‐value
Age at RT, years		0.459
<70	14%	
≥70	48%	
Patient sex		0.361
Female	28%	
Male	52%	
Histology		**0.022**
SCC	52%	
Other	10%	
Stage		0.575
I/II	45%	
III	29%	
Chemotherapy		0.553
No	44%	
Yes	28%	
RT type		0.686
Proton	39%	
Photon	20%	
RT fractionation		0.723
Standard fractionation	29%	
SBRT	50%	
IgG response		0.285
Absent	45%	
Present	14%	
IgM response		0.718
Absent	38%	
Present	26%	
IgG or IgM response		0.602
Absent	39%	
Present	29%	

RT, radiation therapy; SBRT, stereotactic body radiation therapy; SCC, squamous cell carcinoma.

As shown in Table 4, there was no difference in 5‐year LC based on IgG and/or IgM RITI response factors (*P* > 0.05).

**Table 4:** Kaplan Meier analysis for local control

Variables	5‐year local control rate (%)	*P*‐value
Age at RT, years		0.906
<70	79%	
≥70	81%	
Patient sex		0.149
Female	71%	
Male	100%	
Histology		0.615
SCC	77%	
Other	86%	
Chemotherapy		0.142
No	68%	
Yes	89%	
RT type		0.715
Proton	81%	
Photon	75%	
RT fractionation		0.286
Standard fractionation	74%	
SBRT	100%	
IgG response		0.921
Absent	79%	
Present	83%	
IgM response		0.340
Absent	89%	
Present	71%	
IgG or IgM response		0.699
Absent	83%	
Present	77%	

RT, radiation therapy; SBRT, stereotactic body radiation therapy; SCC, squamous cell carcinoma.

## Discussion

We found that radiation does induce antihuman antibodies in patients with NSCLC treated with either photon or proton irradiation by measuring new IgG and IgM expression after radiation in response to tumor antigens from lung cancer cell lines. New IgG or IgM binding was successfully detected in 27% and 50% of patients, respectively. Nesslinger *et al*., using a similar western blotting technique for IgG antibodies, has previously shown that external‐beam RT and brachytherapy for nonmetastatic prostate cancer were associated with the development of treatment‐associated autoantibody responses in 14% and 25% of patients in their study, respectively.^9^ Additionally, our group previously investigated the effect of RT (median 50 Gy to a metastatic site given 10 Gy/fraction) on inducing RITI in 51 patients with metastatic breast cancer treated between 2001 and 2007. IgM or IgG binding was detected in 50% and 57%, respectively (unpublished data).

We also aimed to examine if there was a difference in RITI response in patients treated with proton RT compared to photon RT. Although often assumed to be a low LET treatment, the LET of protons is heterogeneous, with values 10 to 100 times that of photons over the last 2 mm of the beam range (5 to 20 keV/um) at the edge of the spread‐out Bragg peak (SOBP).^13^ High LET has the potential to intensely damage regions of the cell membrane and its proteins, theoretically leading to a more robust antigen. Therefore, tumor cells at the edge of the SOBP may experience an enhanced rate of apoptosis due to the high LET that results in a higher ionization density within the cellular structure compared with the lower LET of photon radiation.^14^, ^15^, ^16^ Thus, we hypothesized that proton therapy may be a better upregulator of RITI compared with conventional photon radiotherapy. Although our limited patient cohort could not detect small differences, we found no large difference in IgG and/or IgM RITI response in patients treated with photon RT compared to patients treated with protons (*P* > 0.05). In our cohort, there were only five patients who received photon RT and 21 who received protons, limiting our ability to detect a statistically significant difference in IgG and/or IgM RITI response based on RT type. Conversely, as the overall RBE of proton therapy is 1.1 and thus the biological effect is similar to that of photons, the higher LET at the end of the SOBP (representing a small volume of the tumor overall) may not result in more robust antigen production, supporting our finding of no difference in RITI‐response. To our knowledge, this is the first study to examine this question in patients with NSCLC treated with RT. Lupu‐Plesu *et al*. recently published results of an *in vitro* study in head and neck squamous cell carcinoma cells examining the biological effects of proton versus photon RT for genes involved in anti‐tumor autoimmunity, namely PD‐L1, and found that both proton and photon RT augment PD‐L1 mRNA expression in a dose‐dependent manner.^17^ Given our limited cohort size, this question should be addressed in future larger studies since no conclusion can be drawn at this time.

In our patient cohort, there was no statistically significant difference in OS, MFS, or LC based on IgG and/or IgM RITI response (*P* > 0.05). Interestingly, in a study of patients with nonmetastatic prostate cancer who received external‐beam RT with neoadjuvant and concurrent androgen deprivation therapy, performed as a follow‐up to Nesslinger *et al.,* 9 patients who developed autoantibody responses to tumor antigens had a significantly lower 5‐year biochemical failure‐free survival rate compared to patients who did not develop an autoantibody response.^18^ On the other hand, our group previously found that for patients with metastatic breast cancer treated with SBRT, the 3‐year OS rate in patients who were IgM‐ or IgG‐positive was significantly better than for those who were negative (unpublished data). This finding suggests that perhaps a large RT fraction size may be necessary to induce an RITI response that is correlated with clinical outcomes, and this warrants further investigation in the future. Higher sized fractions of RT dose have been associated with improved cell‐mediated antitumor immune responses.^19^ Four patients received SBRT and all were in the photon group, potentially explaining the lack of difference between photons and protons. The impact of SBRT versus standard fractionation, and the incremental impact of protons on antitumor immunity requires further investigation.

Using a published approach, tumor antigens from eight different human lung cancer cell lines were used, but patient‐specific tumor antigens may be needed to induce an RITI and therapeutic responses. It was not feasible to obtain primary tumor specimens or serial biopsies to confirm the immune response. The development of RITI was assessed up to 3 months after RT, and most patients were followed until death. It is possible that for some patients RITI may not become evident until a later time‐point, as Nesslinger *et al*. noted the development of treatment‐associated IgG responses within 4 to 5 months of initiation of RT in their study, and IgM was not measured in that study.^9^

There are several notable limitations of our study. While patients who received chemotherapy and those who did not receive chemotherapy were included, the sample size for this preliminary study was too small to perform any formal statistical analyses stratifying patients by receipt of chemotherapy. Future studies should therefore include a group of patients treated with chemotherapy alone without radiotherapy, as well as a group of nontreated patients. Our study used the patient pre‐RT plasma to serve as a baseline and also utilized cell lysates from different lung cancer histologies (adenocarcinoma vs. squamous cell carcinoma) to serve as a negative control, as demonstrated in Fig. 1. Additionally, western blots should be performed in triplicate along with an independent second method for validation of the results in future, larger studies, which was a limitation of this initial preliminary study.

The primary purpose of this study was to determine if radiation induces antitumor antibodies. Indeed, both new IgG and IgM were found in many patients’ blood following radiation consistent with that hypothesis. This observation confirms previous studies in prostate cancer examining for IgG. Notably, it is not possible in our study to determine if these new antibodies were therapeutic or if there was an associated antitumor cellular immune response. In future, however, it will be interesting to perform reverse genetics to discover actionable antigens, using samples from patients with unusually good outcomes. We were underpowered to test our secondary hypotheses to determine if antibody response is associated with improved outcomes or if protons induce a different response than photons.

In conclusion, RITI has been consistently demonstrated across three tumor types in clinical studies: prostate cancer,^9^ breast cancer (unpublished data), and in our study in NSCLC. Nevertheless, there have been mixed results regarding a correlation between RITI response in clinical outcomes, as prostate cancer patients who developed autoantibody responses to tumor antigens experienced worse clinical outcomes^18^ and, in our preliminary study, no correlation with clinical outcomes was detected. However, future studies, with larger sample size, will be needed to determine if RITI correlates with clinical outcomes in patients with NSCLC.

## Disclosure

BH is on the scientific advisory board of Merck & Co., Inc.
